# Host and Viral Proteins Modulating Ebola and Marburg Virus Egress

**DOI:** 10.3390/v11010025

**Published:** 2019-01-03

**Authors:** Tamsin B. Gordon, Joshua A. Hayward, Glenn A. Marsh, Michelle L. Baker, Gilda Tachedjian

**Affiliations:** 1 Health Security Program, Life Sciences Discipline, Burnet Institute, Melbourne, VIC 3004, Australia; tamsin.gordon@burnet.edu.au (T.B.G.); joshua.hayward@burnet.edu.au (J.A.H.); 2 Department of Microbiology, Monash University, Clayton, VIC 3168, Australia; Glenn.marsh@csiro.au; 3 CSIRO Australian Animal Health Laboratory, Health and Biosecurity Business Unit, Geelong, VIC 3220, Australia; Michelle.Baker@csiro.au; 4 Department of Microbiology and Immunology, The University of Melbourne, The Peter Doherty Institute for Infection and Immunity, Melbourne VIC 3010, Australia; 5 School of Science, College of Science, Engineering and Health, RMIT University, Melbourne, VIC 3000, Australia

**Keywords:** filovirus, Ebola virus, Marburg virus, egress, budding, VP40, ESCRT, ubiquitination, viral inhibition

## Abstract

The filoviruses *Ebolavirus* and *Marburgvirus* are among the deadliest viral pathogens known to infect humans, causing emerging diseases with fatality rates of up to 90% during some outbreaks. The replication cycles of these viruses are comprised of numerous complex molecular processes and interactions with their human host, with one key feature being the means by which nascent virions exit host cells to spread to new cells and ultimately to a new host. This review focuses on our current knowledge of filovirus egress and the viral and host factors and processes that are involved. Within the virus, these factors consist of the major matrix protein, viral protein 40 (VP40), which is necessary and sufficient for viral particle release, and nucleocapsid and glycoprotein that interact with VP40 to promote egress. In the host cell, some proteins are hijacked by filoviruses in order to enhance virion budding capacity that include members of the family of E3 ubiquitin ligase and the endosomal sorting complexes required for transport (ESCRT) pathway, while others such as tetherin inhibit viral egress. An understanding of these molecular interactions that modulate viral particle egress provides an important opportunity to identify new targets for the development of antivirals to prevent and treat filovirus infections.

## 1. Introduction

Ebola virus and Marburg virus are among the deadliest viral pathogens known to infect humans, being transmittable primarily through contact with infectious bodily fluids [1]. These viruses can be transmitted between humans and from non-human hosts. The latter include non-human primates, as well as African fruit bats, which are the known and suspected natural reservoir of Marburg and Ebola, respectively [2,3]. Both viruses are members of the family *Filoviridae* of the order *Mononegavirales.* The *Ebolavirus* genus comprises five species that are named after the regions in which they were first observed. These viruses are Bundibugyo virus (BDBV; species *Bundibugyo ebolavirus*); Ebola virus (EBOV; species *Zaire ebolavirus*); Sudan virus (SUDV; species *Sudan ebolavirus*); Tai Forest virus (TAFV; species *Tai Forest ebolavirus*) and Reston virus (RESTV; species *Reston ebolavirus*), with the newly discovered Bombali virus (BOMV; species *Bombali ebolavirus*) currently unclassified but may form a sixth genus [4]. The genus *Marburgvirus* consists of a single species, *Marburg marburgvirus*, and is comprised of two strains, Marburg virus (MARV) and Ravn virus (RAVV), which are approximately 20% divergent at the amino acid level [5]. EBOV and MARV are responsible for some of the most devastating infectious diseases, with case fatality rates reaching up to 90% during some outbreaks [6]. The 2013–2016 outbreak of EBOV in West Africa brought filoviruses into public awareness as an international public health concern, with the Centers for Disease Control and Prevention of the United States reporting over twenty-eight thousand cases and eleven thousand fatalities. There is currently no approved cure or commercially available vaccine for any filovirus. The most promising Ebola vaccine candidate, a recombinant vesicular stomatitis virus-Zaire Ebola virus (VSV-EBOV) vaccine, was approved for emergency use in Guinea in 2016, and the Democratic Republic of Congo in 2017–2018 [7]. Phase III trial results of this vaccine demonstrated an estimated efficiency of ~100% when contacts and contacts-of-contacts were vaccinated as soon as possible [7]. Several other treatments and vaccines are in the earlier stages of development, including a monoclonal antibody cocktail, ‘Zmapp’, and a chimpanzee adenovirus-derived vaccine, ‘cAd3-ZEBOV’, which are undergoing Phase II and Phase III trials, respectively [8,9].

The replication cycle of filoviruses is complex, and involves several viral and host proteins and cellular processes. One of the key features of this replication cycle is the means by which nascent virions exit the host cell in order to infect new cells, and ultimately spread to new individuals. Here we review current knowledge focusing on the role of filoviral proteins in driving viral particle egress and the host cell proteins that either promote, through ubiquitination, viral particle assembly and calcium mobilisation, or suppress this process. An understanding of the molecular interactions relevant to viral particle egress is important to identify new targets for the development of antivirals to prevent and treat filovirus infections.

### 1.1. Filoviral Particle and Genome Structure: A Brief Overview

**Figure 1:**
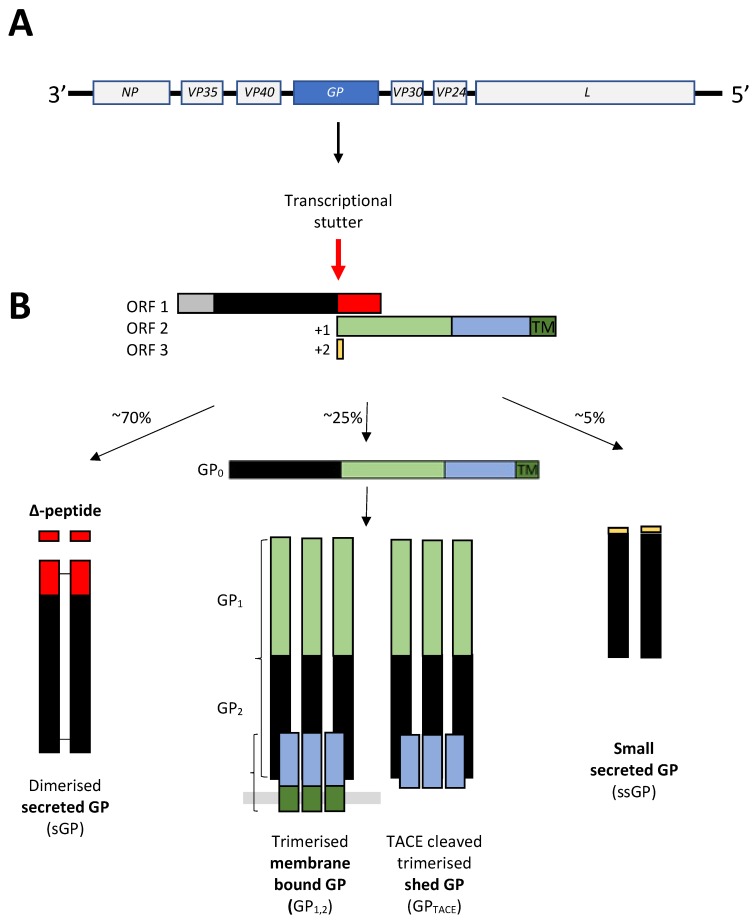
**Ebolavirus glycoprotein.** (**A**) The Ebolavirus genome comprises 7 genes that encode the nucleoprotein (NP), viral proteins 35 (VP35), 40 (VP40), 30 (VP30), and 24 (VP24), the polymerase (L), and four glycoproteins (GP), each transcribed from monocistronic RNA. (**B**) The *GP* gene encodes four products, three of which are generated by transcriptional stuttering at a centrally located polyuridine (poly-U) domain that results in the use of three different open reading frames (ORF1, ORF2 and ORF3). These products (sGP, GP_1,2_, GP_TACE_, ssGP) are produced at the frequencies noted next to the arrows in part B.

Filoviruses are enveloped viruses with a negative-sense, single-stranded RNA genome of approximately 19 kb in length [10]. The virion is filamentous in morphology, and the viral RNA genome is strongly complexed with the nucleoprotein (NP) and viral protein 30 (VP30), which, along with viral protein 35 (VP35) and the L-polymerase (L) protein, form the central nucleocapsid core [11]. Surrounding this structure is a matrix, composed of viral protein 40 (VP40) and viral protein 24 (VP24) [10] and a host-derived lipid envelope in which the glycoprotein (GP) is anchored [10]. The filovirus genome encodes the viral proteins NP, VP35, VP40, VP30, VP24, L and several forms of GP, each transcribed from monocistronic RNA (Figure 1A). Transcription from the filovirus genome is initiated at the 3’ terminal promoter with the individual genes being sequentially transcribed, resulting in more abundant transcription of genes located near the 3’ terminus [12,13]. In the case of EBOV, the GP gene encodes four products through transcriptional stuttering at a centrally located polyuridine (poly-U) domain [14] (Figure 1B). These four GP proteins are the GP_1,2_ surface protein, cleaved from the full length GP_0_ precursor by furin-like proteases into GP_1_ and GP_2_, which assemble into the virion lipid envelope as 450 kDa trimers [15,16]; a secreted version of the GP_1,2_ trimer lacking the transmembrane domain (GP_TACE_) [17]; a 110 kDa secreted form of GP (sGP) that forms dimers via a unique C-terminal domain [14]; and a 100 kDa truncated small, secreted GP (ssGP) that is also dimeric [18,19]. In addition, there is a 5 kDa secreted Δ-peptide representing the C-terminal cleavage product of pre-sGP [11] (Figure 1B).

### 1.2. Filoviruses Bud from the Host Cell to Complete the Replication Cycle

**Figure 2:**
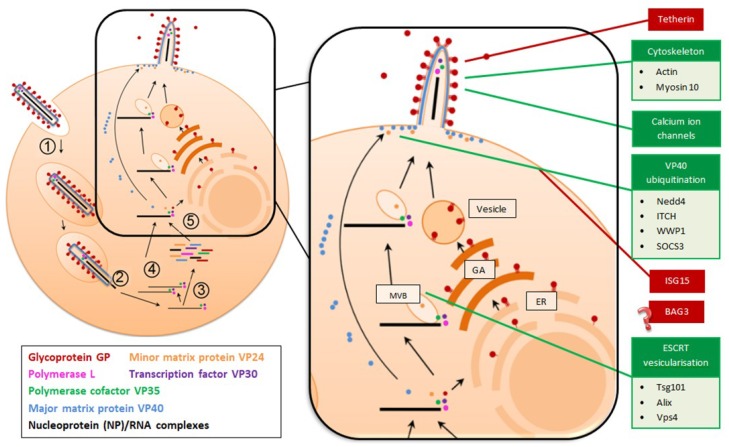
Filovirus replication cycle showing host and viral proteins that either promote (green boxes) or block (red boxes) virion assembly and release. Following viral attachment and entry into the host cell (1), the nucleocapsid, containing genomic RNA, is released into the host cell cytoplasm (2) and transcription of filovirus genes (3) and genome replication occurs. Replication is catalysed by the polymerase and viral cofactors NP, VP30 and VP35 (4). In the magnified diagram (5), nucleocapsids are transported to the plasma membrane by hijacking endosomal sorting complexes required for transport (ESCRT) machinery and the multivesicular body (MVB) trafficking pathway. Simultaneously, the major matrix protein, VP40, oligomerises as it moves towards the plasma membrane, while the full length glycoprotein is sent to the endoplasmic reticulum (ER) and Golgi apparatus (GA) for processing and is subsequently transported in vesicles to the plasma membrane in its trimeric form. Nascent virions assemble at lipid rafts and bud from the host cell. In the case of Marburg virus, budding occurs from actin filopodia. Host factors that promote viral egress (green boxes) include actin and myosin, calcium ion channels, neural precursor cell expressed developmentally down-regulated protein 4 (Nedd4), Itchy E3 ubiquitin protein ligase (ITCH), WW domain-containing E3 ubiquitin protein ligase 1 (WWP1), suppressor of cytokine signaling 3 (SOCS3), tumor susceptibility gene 101 (Tsg101), ALG-2-interacting *protein* X (Alix), and vacuolar protein sorting 4 (Vps4). Host factors that interfere with viral egress (red boxes) include tetherin, interferon stimulated gene 15 (ISG15) and BCL2 associated athanogene 3 (Bag3).

The process of filovirus entry is complex, with the processes and cellular factors involved yet to be fully elucidated. Filovirus replication is initiated by GP_1,2_ binding to a range of cellular carbohydrate binding receptors, and interactions between phosphatidylserines lipids in the viral envelope and cellular phosphatidylserine receptors [20]. After binding, virions are internalised into vesicles, primarily through macropinocytosis [21] (Figure 2), involving complex lipid-mediated signalling pathways. Viral nucleocapsids are eventually released into the cell cytoplasm, requiring the cooperation of a number of factors, including endosomal acidification, GTPase activity, the presence of various endosomal markers and use of calcium ion channels [20]. Endosomal acidification also promotes removal of the GP_1,2_ glycan cap [22] by host cathepsins [23,24,25], subsequently exposing the receptor binding domain [26] that is required for binding to the critical intracellular receptor Niemann-Pick C1 [27]. While these events are all necessary stages in the fusion process, they are not by themselves sufficient to induce the fusion event with the endosomal membrane that gives nucleocapsids access to the cell cytoplasm. Transcription of filovirus genes and genome replication occurs within the nucleocapsid complex and is catalysed by the RNA-dependent RNA polymerase activity of the L polymerase in concert with NP and the VP30 and VP35 cofactors [28,29]. The resulting positive-sense mRNAs are subsequently capped and polyadenylated [28,29] before being translated into structural and non-structural proteins at cellular ribosomes. Genome replication occurs in cytoplasmic inclusion bodies [30], which also act as the site of nucleocapsid assembly [31]. Nucleocapsids and GP_1,2_ are independently transported to the plasma membrane, nucleocapsids by hijacking endosomal sorting complexes required for transport (ESCRT) machinery and the multivesicular body (MVB) trafficking pathway [31] and GP in vesicles, following processing in the endoplasmic reticulum (ER) and Golgi apparatus (GA) [32]. VP40 assembles into dimers and subsequently into hexamers as it also moves towards the plasma membrane.

The final process of filovirus replication is the budding of viral particles from the host cell. VP40, the filoviral major matrix protein, is the most abundant of the structural proteins [33] and is necessary and sufficient for driving viral particle formation [34,35]. VP40 links the nucleocapsid to the cytoplasmic side of the cell membrane and facilitates the budding event that completes the viral replication cycle [36]. Efficient viral egress from host cells relies on complex interactions between viral and host proteins, often involving VP40.

## 2. Viral Proteins that Promote Filovirus Budding

### 2.1. Central Role of the VP40 Matrix Protein in Filoviruses Egress

The VP40 matrix protein is the major structural protein responsible for viral egress. This is due to VP40 being necessary and sufficient to promote the production of virus-like particles (VLPs) from host cells [37,38]. The ability of VP40 to form viral particles is dependent on its oligomerisation state, binding to the plasma membrane, and the presence of key internal motifs. These processes and structures will be briefly addressed in order to provide an overview of the main mechanism by which filovirus egress occurs, providing the context for subsequent discussion of the role of host and viral proteins that directly or indirectly interact with VP40 to influence the efficiency of filovirus egress.

#### 2.1.1. VP40 Late Domain

**Figure 3:**
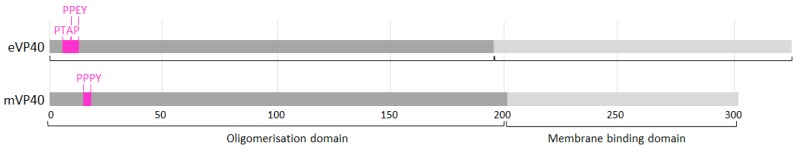
Domain organisation of EBOV and MARV VP40. VP40 is composed of a C-terminal membrane binding domain and an N-terminal oligomerisation domain. The late (L) domain PTAP and PPEY motifs are denoted in pink. Motifs were obtained from EBOV VP40 (eVP40) and MARV VP40 (mVP40) uniprot database entries (Q05128 and Q1PD51, respectively).

VP40 contains two distinct domains, a C-terminal membrane-binding domain and an N-terminal domain responsible for oligomerisation and budding (Figure 3) [39]. Contained within the N-terminal domain is a late domain (L-domain), which for EBOV is necessary for efficient budding [40]. The EBOV VP40 (eVP40) L-domain is unique, harbouring overlapping PTAP and PPxY motifs arranged as PTAPPEY [41]. Deletion of any single L-domain motif of eVP40 is sufficient to abrogate budding [42]; viral particle and overall replication capacity is dramatically decreased when both motifs are mutated [43,44]. In contrast to eVP40, the MARV VP40 (mVP40) L-domain contains a PPxY, but no PTAP, motif. The PPxY motif, whilst not essential for VLP egress, improves budding efficiency by up to 70% [45,46].

These proline-rich motifs, found in L-domains, bind to WW domains (also known as rsp5-domains or WWP repeating motifs). WW domains are unusually small, being composed of approximately 40 amino acids [47], and are named for their two conserved tryptophan (W) residues, which are separated by 20–22 amino acids [47]. Notably, a wide range of proteins have been identified as containing WW domains, including several host proteins such as neural precursor cell expressed developmentally down-regulated protein 4 (Nedd4), Itchy E3 ubiquitin protein ligase (ITCH) and BCL2 associated athanogene 3 (Bag3). These proteins interact with the L-domain of eVP40 via their WW-domain and in so doing impact the efficiency of viral budding [48,49,50], through a number of different mechanisms which will be discussed in various contexts in Section 3 and Section 4 of this review.

#### 2.1.2. VP40 Oligomerisation and Membrane Binding Motifs

Localisation and attachment of VP40 to the plasma membrane is necessary for filovirus budding and depends on the formation of VP40 antiparallel dimers and subsequent higher order oligomerisation [51]. Wild-type eVP40 forms stable linear hexamers and octamer rings [52] depending on the interdimeric interface that is utilised. Octameric eVP40 is necessary for binding and incorporation of genomic RNA into nascent VLPs [53,54], whereas hexameric eVP40 has a role in lipid raft formation and is necessary for viral particle formation [55,56]. Several amino acid residues have been identified as being necessary for efficient oligomerisation of eVP40 [40,57,58,59,60] and therefore for efficient egress (Table 1). Amino acids have been similarly identified in mVP40, which, when mutated, prevent oligomerisation and budding [39,46]. In addition, alanine mutagenesis studies have identified a proline at position 53 in the C-terminal domain that is essential for localisation of VP40 at the plasma membrane and subsequent budding from the cell [57], as are a number of additional amino acids [60,61] (Table 1).

Oligomerisation of VP40 dimers occurs at lipid rafts in the host cell plasma membrane [64], which are characterised by a higher than average concentration of cholesterol and liquid-ordered glycosphingolipids. Cholesterol levels do not have any effect on EBOV budding efficiency [68]. However, increasing levels of cholesterol in the plasma membrane positively affects MARV budding through mVP40 association, possibly as a result of the “cholesterol condensation effect” [69,70]. This effect is defined as increasing cholesterol levels in a lipid membrane, resulting in tighter packing of the phospholipid molecules, including those to which VP40 binds, than what would normally be expected [71]. The lipid composition of rafts is critical for efficient binding of both mVP40 and eVP40 to the plasma membrane. Phosphatidylserine is the most highly concentrated anionic lipid in the cytosolic leaflet of the cellular membrane and is primarily responsible for interactions with VP40, as it is for interactions with viral matrix proteins of other viruses, including HIV-1 Gag [39,58,70,72]. eVP40 is unable to localise to the plasma membrane of PSA-3, a CHO (Chinese hamster ovary) mutant cell line with approximately 30% less plasma membrane phosphatidylserine than is normally present [73]. This suggests that a high threshold concentration of phosphatidylserine is required for matrix protein-plasma membrane interactions. Additionally, no eVP40 binding is observed in vesicles that model the plasma membrane with a phosphatidylserine content of below 8 mol% [73]. This effect is not reversed by the replacement of phosphatidylserine with other anionic lipids [68]. Taken together, these data highlight the importance of plasma membrane phospholipid composition for the assembly of filoviral particles.

Although eVP40 and mVP40 are only 34% identical and 49% similar by amino acid sequence [39], they share many of the structural elements required for binding to the plasma membrane. eVP40 binds to the basolateral side of the phosphatidylserine rich plasma membrane via two cationic patches in its C-terminal domain (residues K224/K225 and K274/K275 as shown in Table 1) [39,74,75]. When dimerised, the cationic patches of eVP40 monomers come together to form a larger cationic domain, which therefore has enhanced avidity for the plasma membrane and results in preferential binding of dimerised over monomeric eVP40 [75]. The mVP40 cationic patch is more extensive than that of eVP40, comprising of two instead of one basic loop structure; however, mVP40 dimerisation is still necessary for membrane association [39]. It has been demonstrated for eVP40 that interactions with phosphatidylserine promotes eVP40 hexamerisation, allowing docking to the cell membrane via a C-terminal hydrophobic loop (residues I293 through A299 as shown in Table 1) [67]. Tight packing and oligomerisation of eVP40 at lipid rafts significantly lowers the thermal energy requirement to bend the membrane in the process of virion formation [76].

Although phosphatidylserine is the only lipid that has been identified as critical for eVP40 binding to the plasma membrane, proper localisation and extensive oligomerisation requires phosphatidylinositol 4,5-biphosphate (PI(4,5)P_2_), which is critical for HIV-1 budding [77,78]. Phosphatidylserine and PI(4,5)P_2_ perform distinct roles in eVP40 oligomerisation. Phosphatidylserine is responsible for the initial hexamerisation of eVP40 dimers [77], while eVP40 hexamers subsequently enhance clustering of PI(4,5)P_2_, which in turn controls the formation of oligomers 12-mer and greater, possibly through a stabilising mechanism [56,77]. Interactions between PI(4,5)P_2_ and eVP40 are much weaker in the absence of phosphatidylserine; this supports evidence that prior interactions with phosphatidylserine are needed for subsequent PI(4,5)P_2_ binding [77]; however, the details of this process of eVP40-lipid interaction, and membrane insertion and oligomerisation, remain unclear. In contrast to eVP40, anionic phospholipids other than phosphatidylserine, such as phosphatidylinositol derivatives, have a charge-dependent but non-specific additive effect on mVP40 association with the plasma membrane [70]. The role of PI(4,5)P_2_ on mVP40 interactions with the lipid membrane has been examined through its selective depletion (63). These experiments confirmed the promiscuity of mVP40 compared to eVP40, showing no preference for binding to PI(4,5)P_2_ compared to other lipids [70].

### 2.2. GP and NP are Promoters of VP40 Egress

While VP40 is the primary driving force behind filoviral budding, other viral proteins, such as GP and NP, contribute to efficient VLP release. Co-expression of EBOV GP_1,2_ (eGP) with eVP40 increases VLP budding by 5–8 fold [13]. Similar to EBOV, co-expression of mVP40 and MARV GP (mGP) results in increased budding efficiency of MARV VLPs [79]. Expression of eGP also partially negates the reduction of budding efficiency resulting from deletion of the eVP40 L-domain [59]. The influence of eGP on budding is specific to membrane bound eGP, as neither glycoprotein from vesicular stomatitis virus (VSV) nor TACE protease-cleaved GP (GP_TACE_) affect EBOV budding [13].

Co-expression of EBOV NP (eNP) with eVP40 also increases budding efficiency of EBOV VLPs [13]. Budding efficiency and incorporation of eNP into eVP40 VLPs are negated when the C-terminal domain of eNP is deleted [13]. This suggests that budding promotion by eNP requires the protein to first be recruited to the plasma membrane by eVP40, as the C-terminal domain is responsible for membrane localisation and binding. When eVP40, eGP and eNP are co-expressed in cells, an additive effect is observed, resulting in an overall increase in maximum budding efficiency of greater than 40-fold compared to eVP40 alone [13]. Similarly, efficiency of mVP40 VLP budding is significantly increased by the expression of MARV NP (mNP) [80]. Notably, mNP contains an N-terminal PTAP motif and a C-terminal PSAP motif [81]. Although mutation of the PTAP motif has little impact on the positive effect on VLP release, mutation of PSAP alone or combined with a mutation in PTAP, almost entirely abrogates the positive effect that mNP has on egress, indicating that it plays a critical role in the promotional role of mNP on VLP egress [81]. No such PSAP/PTAP motifs are present in eNP [81], indicating that its role in this context must depend on an alternative mechanism.

## 3. Host Cell Proteins that Promote Filovirus Budding

Filoviruses have developed several strategies to hijack host proteins to promote replication in the cell. These host proteins include cytoskeletal proteins that are critical for the formation of filopodia, the site of MARV virion egress, several ubiquitin ligases that ubiquitinate VP40 and help target virions to the plasma membrane for budding, and ESCRT proteins that mediate the budding process. In addition, calcium ions support the function of calcium-dependent molecules that act as accessory proteins to ubiquitin ligases and ESCRT proteins to promote virion egress.

### 3.1. Hijacking of the Host Cytoskeleton Facilitates Filovirus Budding

Both EBOV and MARV hijack the host cytoskeleton to facilitate assembly and budding of their virions [63,82,83,84,85], as observed for other enveloped viruses such as HIV [86,87,88]. In the case of EBOV, the host cytoskeleton is hijacked in order to aid in trafficking components of the new virions to the site of budding, with three-dimensional live tracking imaging showing that nascent VLPs are associated with actin as they approach the plasma membrane [85]. However, in the case of MARV, actin is more directly involved in efficient budding, with the majority of budding VLPs (86.3 ± 7.6%) being associated with filopodia [84]. The interaction between actin filopodia and mVP40 VLPs has been observed through immunofluorescence assays, with mVP40-containing bodies and actin bundles co-localising in filopodia which extend from the plasma membrane [89]. This effect has also been observed through transmission electron microscopy of monolayers of MARV-infected cells [84]. In MARV VLPs, actin is found clustered at a single point in the viral envelope, directly overlapping with the portion of the viral membrane that attaches to actin filaments prior to budding [84]. Use of actin-depolymerising drugs, cytochalasin-D and latrunculin-B, results in significant decreases in the number of VLPs and of infectious viral particles that are released from the cell surface [84]. Similarly, overexpression of host promoters of filopodia assembly, cell division cycle 42 (Cdc42) and myosin 10, is associated with increased efficiency of MARV VLP budding [84]. Correct localisation and budding of MARV VLPs is significantly reduced when the cargo binding tail of myosin 10, which is found at the tip of actin filaments, is deleted [84]. These data suggest that the interaction between MARV and actin requires that myosin 10 acts as an intermediate, binding to mVP40 and actin simultaneously to facilitate their indirect interaction.

### 3.2. VP40 Ubiquitination is Necessary for Filovirus Budding

Filoviruses hijack members of the family of E3 ubiquitin ligases, which function broadly in targeting poly-ubiquitinated proteins for degradation by proteasomes, or mono-ubiquitinated proteins to the cell surface for recycling [90]. Ubiquitin ligases are utilized by filoviruses to mono-ubiquitinate [91] VP40 and therefore target it for transport to the cell surface where virion assembly and budding then occurs [92]. Transportation is mediated by the ESCRT pathway, which is discussed in Section 3.3. It has not been clarified in the literature whether or not ubiquitin ligases use the L-domains as the site of ubiquitination of VP40; the online ubiquitination predictor UbPred predicts K236 and K326 in eVP40 and K259 in mVP40 as low to moderate likelihood sites of ubiquitination; however, these sites have not been mentioned in the literature in the context of ubiquitination. These sites may be worthy of investigation in the future.

#### 3.2.1. Nedd4

The E3 ubiquitin ligase most widely researched in the context of filovirus budding is Nedd4. Nedd4 is normally localised to the perinuclear region but can become associated with lipid rafts at the cytoplasmic membrane [93,94]. The protein has a modular structure that is a characteristic of all Nedd4-like E3 ubiquitin ligases, consisting of a membrane binding domain, four central WW domains, which bind to PPxY motifs in target proteins (e.g., eVP40), and a C-terminus homologous to the E6-AP Carboxyl Terminus (HECT) ubiquitin ligase domain [48].

Interactions between eVP40 and Nedd4, as determined by coimmunopreciptation studies, are mediated though the PPxY domain of eVP40 and the WW domain of Nedd4 [45,95]. A study of Nedd4 that introduced tryptophan-to-glycine mutations to each of the WW domains showed that distinct WW domains facilitate the interaction of Nedd4 with eVP40 from different filoviruses, with mVP40 binding most strongly with WW domain 1 and eVP40 with WW domain 3 [45]. This binding preference of eVP40 compared to mVP40 has been hypothesised as being due to differences in the regions flanking the PPxY domains of eVP40 and mVP40 [62]. Mutation of any single WW domain is not sufficient to prevent Nedd4 interactions with eVP40 [95], suggesting that WW domain 3 is preferentially used, but not essential in this context. In the case of both EBOV and MARV, the interaction between Nedd4 and VP40 results in increased egress of VLPs [45,95].

Enhanced filovirus VLP budding in the presence of Nedd4 is most likely modulated by a mono-ubiquitination event catalysed by Nedd4, as EBOV VLP release is reduced by 44% by mutation of the HECT domain compared to wild-type Nedd4 [95]. This HECT mutation also has a similar mitigating effect in the context of MARV budding [45]. Interestingly, mutation of the C2 domain of Nedd4 enhances the budding efficiency of EBOV VLPs, possibly due to changes in Nedd4 protein conformation that allow for better access to the WW domain by eVP40 [95]. The same enhancing effect is not observed in the case of mVP40 [45].

#### 3.2.2. ITCH

Itchy E3 ubiquitin protein ligase (ITCH), also known as atrophin-1 interacting *protein* 4 (AIP4), is a Nedd4-like ubiquitin ligase E3 protein that localises to the perinuclear space and endocytic vesicles [96]. It is one of nine identified Nedd4-like E3 proteins [97], demonstrating the strongest binding to eVP40 [49]. ITCH was identified as an eVP40 interacting partner in a screen of proteins containing a proline-rich WW or SH3 (src homology 3) domain that demonstrate complementarity to the PPxY domain. ITCH, which like Nedd4 contains four central WW domains, interacts with eVP40 PPxY exclusively through a highly specific interaction with its first WW domain, indicating a potential biological role for ITCH in EBOV release [49]. The interaction between ITCH and eVP40 results in the mono-ubiquitination of eVP40 and enhancement of VLP egress by a factor of 2 to 3-fold [49]. Conversely, knockdown of ITCH through the application of ITCH-specific siRNAs results in a 75–80% reduction in EBOV VLP egress, which is reversed by transiently reintroducing ITCH to the system [49]. These observations were also confirmed with infectious virions, underscoring a role for ITCH in EBOV budding. ITCH interactions with mVP40 have not been explored.

In the context of independent viral infection, ITCH ligase activity is controlled through a number of intramolecular and autoinhibitory interactions that aid the regulation of the ubiquitination cascade. The WW domains of ITCH are critical to a number of these interactions. c-Jun N-terminal kinase (JNK)-mediated phosphorylation of ITCH is essential for its ligase function, with JNK1 interacting with ITCH at a region neighbouring the first WW domain [98]. Accordingly, a selective inhibitor of JNK, SP600125, has been utilised to examine the relevance of JNK in the context of EBOV. Budding of EBOV VLPs is reduced by ~10- and 20-fold in the presence of 5 and 10 μM of SP600125, respectively, suggesting that activation by JNK is necessary for the positive influence of ITCH on the viral replication cycle [49]. Although the potential consequences of eVP40 interactions with ITCH on its regulatory functions are yet to be fully elucidated, the evidence that JNK interactions with ITCH influences eVP40 binding suggests that eVP40 binding does not interfere with the interaction of JNK to its WW domain 1-neighboring binding site.

#### 3.2.3. WWP1

WW domain-containing E3 ubiquitin protein ligase 1 (WWP1), also known as AIP5 (ALG-2-interacting *protein* 5), is another Nedd4-like E3 protein found to promote EBOV egress as a result of interactions with eVP40 [91]. WWP1 contains four WW domains, and while all four are able to interact with the eVP40 PPxY motif, the WW1 is the most critical for the interaction with eVP40 [91]. Confocal microscopy demonstrates that WWP1 colocalises with eVP40 at the plasma membrane [91]. Mutation of the active ubiquitination site or knockout of WWP1 decreases expression of mature, mono-ubiquitinated eVP40, VLP production and budding efficiency [91]. Collectively these data underscore the biological relevance of WWP1 in eVP40 egress. No data is available addressing the effect of WWP1 on MARV budding.

#### 3.2.4. SOCS3

Although different in general structure from the Nedd4-like E3 ubiquitin ligases, suppressor of cytokine signaling 3 (SOCS3) is also involved in the ubiquitination process, functioning in part as a recruiter and component of a newly identified class of Cullin5-based E3 ubiquitin ligase complexes [99,100]. SOCS3 expression is amplified by the mucin domain of eGP, indicating that SOCS3 may have a specific biologically relevant role in EBOV infection [101,102]. Similar to Nedd4 and ITCH, SOCS3 ubiquitinates eVP40 and enhances the egress of both VLPs and infectious virions by a factor of 3–4 fold [101]. SOCS has not been explored in the context of MARV egress. SOCS3 knockout-mutants decrease the efficiency of EBOV VLP egress, which is reversed by transiently reintroducing SOCS3 [101]. Ubiquitination of eVP40 is mediated by the C-terminal SOCS-box domain [100]. SOCS3-mediated promotion of EBOV egress results in SOCS3 incorporation into EBOV virions; whether virion incorporation of SOCS3 enhances the infection of target cells remains to be determined [101].

### 3.3. The Host ESCRT Pathway Drives Filovirus Egress

The ESCRT pathway drives the formation of vesicles, used in a metabolic context to sort proteins for transportation to various cellular locations [103]. Protein sorting is a highly complex and convoluted process, with cargo reaching its destination by one of several routes. The pathway that proteins are targeted in the cell is in part dependent on their ubiquitination status, where poly-ubiquitination usually serves to target proteins for degradation, while mono-ubiquitinated proteins are often recycled to the cell surface [90]. The ESCRT machinery is made up of four protein complexes (ESCRT-0 through ESCRT-III), composed of class E vacuolar protein sorting proteins, as well as a number of accessory proteins. In the following section, ESCRT proteins that have currently been identified as being hijacked by filoviruses to drive the formation and release of viral particles will be discussed together with their mechanism of action and impact on viral egress.

#### 3.3.1. Tsg101

Both EBOV and MARV hijack the ESCRT machinery to drive their egress from the cell surface, with a number of ESCRT proteins shown to be critical for efficient viral budding. One such protein is tumor susceptibility gene 101 (Tsg101), which is also exploited by other enveloped viruses to drive viral egress [59,104,105,106,107,108,109]. Tsg101 is a component of ESCRT-I, which interacts directly with eVP40 and mVP40 to increase the efficiency of VLP budding [41,80]. Tsg101 is an inactive homologue of an E2 ubiquitin-conjugating protein and its main function in the cell is in vesicularisation, predominantly for the purposes of cell abscission and protein trafficking or degradation [110,111]. eVP40 can alternatively recruit Tsg101 from the endosomes to accrue at the plasma membrane at the site of eVP40 localisation in order to facilitate budding [59,109]. The eVP40-Tsg101 interaction is mediated through the vestigial E2 enzyme active site (UEV binding pocket) in Tsg101 [112]. The eVP40 L-domain mediating this binding interaction has not been confirmed, with mutation of either the PTAP or PPxY motifs yielding contradictory data [41,42,59,109]. In this regard, studies report that the overlapping PPxY and PTAP motifs of eVP40 are functionally redundant [41,42,59]. In contrast, another study reported that mutations to PPxY have no noticeable effect, while mutations to PTAP cause a budding defect of an equivalent magnitude to deletion of the entire eVP40 N-terminus [109]. This contradiction may be due to differences in the experimental design between these studies, with different mutations having been introduced to the L-domain motifs, or may suggest that the binding motifs are flexible and interact with Tsg101 differently depending on small variations in the experimental conditions. The interaction between eVP40 and Tsg101 results in Tsg101 incorporation into VLPs, which for successfully budded EBOV VLPs is dependent on the presence of wild-type eVP40 [41]. A VSV-recombinant model expressing the PPxY domain from eVP40 demonstrates that efficient budding relies on Tsg101 incorporation, with siRNA knockdown of Tsg101 resulting in a 5-fold reduction in EBOV virion release compared to when Tsg101 is present [113].

Tsg101 also promotes MARV egress, with Tsg101 siRNA knockdown significantly reducing the budding efficiency of MARV VLPs by an average of 32% [108]. Tsg101 is incorporated into budded MARV VLPs [80,81], as is also observed for EBOV [95]. However, in contrast to EBOV, mVP40 does not possess a PTAP motif. Mutation of mVP40 L-domain residues identified the PPxY motif as critical for interacting with Tsg101 [80]. Tsg101 also interacts directly with the PSAP domain in the mNP. Localisation of Tsg101 at sites of MARV budding, originally assumed to be mediated by mVP40, does not occur in systems expressing Tsg101 and either mNP or mVP40 [81]. Rather, co-expression of mVP40 and mNP is necessary for Tsg101 localisation to the plasma membrane [81]. Mutation of the PSAP domain in mNP reduces the recruitment and incorporation of Tsg101 into viral particles by 20%, in addition to abolishing the positive effect that mNP has on budding [81,108]. Immunofluorescence assays examining the localisation of Tsg101 and mNP have identified an ability for Tsg101 to influence nucleocapsid transport as part of its role in promoting MARV budding [108].

Tsg101 co-localises with IQ motif containing GTPase activating protein 1 (IQGAP1), a cellular regulator of actin cytoskeleton dynamics, which acts through Tsg101 to recruit mNP via actin-dependent transport to the sites of budding [108]. Knockdown of IQGAP1 by siRNAs demonstrates that this protein plays a critical role in MARV transport to the site of budding, with depletion of the protein reducing viral titres by a factor of 2–3 fold [108]. Additionally, imaging shows IQGAP1 trails behind nucleocapsids as they move towards the plasma membrane, as expected of a propulsion system [108]. A similar effect has been demonstrated in the case of EBOV [114]. To summarise, filoviruses are able to recruit Tsg101 to the cell surface in order to utilise its vesicularising capacity in the process of filovirus budding. The function of Tsg101 in this context is dependent on its interaction with VP40 in the case of EBOV, and VP40 together with NP in the case of MARV, with these interactions increasing the budding efficiency of nascent virions.

#### 3.3.2. Alix

ALG-2-interacting *protein* X (Alix), also known as AIP1, is an ESCRT-associated protein, which, among its many roles, functions as a critical recruiter of ESCRT complexes and associated proteins [115]. Like Tsg101, Alix is well recognised as having a role in promoting the egress of other viruses, particularly in the context of HIV-1 budding [116]. Alix contains three domains: Bro1, V, and PRD, with the latter containing a PPxY motif [117]. Alix is known to have a positive effect on HIV-1 budding, even when the PPxY domain of Gag is mutated [116], due to the presence of the YPx(n)L motif in Gag interacting with the V domain of Alix. Thus, the YPx(n)L motif gives Alix the capability to rescue viral budding in the absence of a functional PPxY domain that interacts with Tsg101 [116]. In the case of EBOV, Alix is able to perform a similar role to that reported for HIV-1 budding. Evidence for such a role in egress is supported by the observation that deletion of the eVP40 PTAPPPEY domain reduces VLP release by only 40% compared to wild-type eVP40, indicating that budding can be mediated by a Tsg101-independent mechanism [59]. Alix-mediated budding may account, in part, for this remaining 60% VLP release defect. Alix enhances budding of EBOV VLPs only when the PPxY domain of eVP40 is mutated, an enhancement that is increased from 2–3 fold to 4–7 fold when the Alix Bro1-V fragment is used in place of the wild-type molecule [118]. This enhancement of eVP40 budding may be as a result of conformational changes in Alix which occur when the proline-rich domain is removed [119], giving eVP40 better access to its binding site. The relevance of Alix on EBOV budding has been confirmed by siRNA knockdown of Alix and by co-immunoprecipitation assays with eVP40 [118]. There has not been any data published on the effect of Alix on MARV budding.

A subsequent search for a motif in filoviruses that interacts with Alix identified YPx(n)L/I in the VP40 N-terminus of all *Ebolavirus* strains except RESTV and TAFV [118]. Mutation of this YPx(n)L/I motif abrogates the enhancing effect of Bro1-V on EBOV VLP budding [118]. In experimental systems where eVP40 harbours mutations in PPxY alone or together with the YPx(n)L/I, Bro1-V is detected only in released VLPs containing a functional YPx(n)L/I domain [118]. This observation confirms that the YPx(n)L/I motif acts as an alternative functional L-domain that interacts with Bro1-V domain of Alix and contributes to budding [118]. In the case of HIV-1, it has been demonstrated that Alix is able to recruit Nedd4 to act as a bridging protein between itself and HIV-1 Gag to promote budding in instances where a defective PPxY domain would otherwise prevent ubiquitination by Nedd4 [120]. Additionally, Nedd4 ubiquitinates Alix, which is necessary for its incorporation into HIV-1 particles [120]. The relevance of Nedd4 in Alix-mediated EBOV budding has not been reported but is worthy of examination.

#### 3.3.3. Vps4

Vacuolar protein sorting 4 (Vps4) is a type I AAA ATPase capable of dissociating class E vacuolar protein-sorting proteins, such as ESCRT-III, from endosomal membranes when assembled into a dodecameric complex by its cofactor, Vta1 [121,122], as well as mediating membrane excision [123]. As in the case of Tsg101, Vps4 is localised to endosomes in the absence of eVP40, whereas in the presence of eVP40 it is re-localised to plasma membrane [59]. As expected, the ATPase activity of Vps4 is necessary for its efficient role in promoting viral budding with enzymatically inactive mutants of Vps4 demonstrating a 60–90% reduction in EBOV VLP egress compared to the wild-type control [41,59]. Vps4 is responsible for a 54% reduction in MARV VLP budding [124].

### 3.4. Free Calcium Ions Promote Efficient Budding of Filoviruses

The many roles of calcium include endosomal membrane fusion and exocytosis, with select calcium-dependent accessory proteins reported to interact with ESCRT proteins, including Tsg101 and Alix, and the E3 ubiquitin ligase Nedd4 [125,126,127,128]. It is therefore not surprising that calcium and the calcium-binding protein calmodulin are associated with the budding of HIV-1 and filoviruses [128,129]. The presence of eVP40 and mVP40 promotes the accumulation of calcium in the intracellular space [130]. The intracellular calcium ion chelator 1,2-bis(*2*-amino-5-phenoxy)ethane-*N*,*N*,*N′*,*N′*-tetraacetic acid in combination with acetoxymethyl ester (BAPTA/AM), was used to demonstrate that decreasing levels of free calcium significantly reduces EBOV VLP budding, both when eVP40 is expressed alone or in concert with eGP [130]. A similar viral budding defect was observed with recombinant VSV engineered to contain the eVP40 L-domain residues [130]. Conversely, egress is significantly enhanced by ionomycin, which recruits calcium into the cytoplasm from the extracellular environment and cytosolic stores [130]. This phenotype has been observed with eVP40 alone and when VP40 is co-expressed with eGP [130]. Recruitment of calcium ions into the cytoplasm is dependent on manipulation of the store-operated calcium entry pathway. This pathway is initiated when calcium levels in the ER become critically low, causing Ca^2+^ ions to dissociate from ER membrane-spanning stromal interaction molecule (STIM) proteins, inducing a conformational change in STIM1 [131]. The conformational change in STIM1 facilitates interactions with Orai1 calcium release-activated calcium channel proteins embedded in the cell envelope [131]. This interaction opens the Orai1 channel, allowing calcium to flood back into the cell to restock ER stores [131]. siRNA-mediated suppression of STIM1 in cells results in a 10-fold reduction in eVP40 VLP budding, with egress being rescued when STIM1 is expressed ectopically [132]. Similarly, single amino acid mutations to Orai1 subunits, and Orai1 inhibitors (Synta66, 2-APB and RO2959) all negate increases in calcium cytoplasmic levels and reduce VLP formation and budding of both eVP40 and mVP40, as well as decreasing the overall viral load and the number of cells subsequently infected [132].

Calcium is involved in a number of signalling pathways, components of which have been inhibited in order to determine whether they have an effect on EBOV budding [130]. These experiments reveal that budding is dependent on the Ras/Raf/MEK/ERK signalling pathway, with inhibitors of calmodulin (W7, trifluoperazine and W13) and MEK (U0126) significantly reducing the budding of both eVP40 alone, and when co-expressed with eGP [130]. These results are anticipated given the identification of a putative ERK phosphorylation site in eVP40 [130].

## 4. Host Inhibitors of Budding

While filoviruses are able to interact with their host in a manner that is to their advantage, host cells have strategies to interfere with viral proteins in order to diminish this advantage. These include tetherin, that tethers nascent virions to the host cell surface, ISG15, that interferes with Nedd4 function, and Bag3, that targets VP40 for autophagy. These proteins may provide foundations on which to design strategies to combat filovirus infections in a prophylactic or treatment context.

### 4.1. Tetherin Antagonises Virion Release from the Host Cell Surface

Tetherin, also known as CD317 and BST-2 (bone marrow stromal antigen 2), is an intracellular restriction factor that is up-regulated in a wide range of human cell types in response to type I IFN [133]. Tetherin was initially identified as a host factor which restricts the egress of HIV-1 from the host cell, and has since been found to target a diverse range of enveloped viruses, including filoviruses [134]. Human tetherin inhibits viral spread by tethering mature virions to the surface of host cells and to each other [135,136,137,138,139] at lipid raft domains at the plasma membrane. Virions are released from this tether by viral proteases [133,140].

Tetherin is a relatively non-polymorphic type II protein [141,142], and is composed of three structural domains: a transmembrane domain, a coiled-coil ectodomain, and a glycosylphosphatidylinositol (GPI) anchor [143,144]. Both terminal domains of tetherin have the capacity to interact with lipid membranes, a topology that is highly unusual [145]. A pivotal study by Perez-Caballero et al. [146] constructed a protein with no sequence homology to native tetherin, but which adopted a similar conformation. This protein was able to effectively tether HIV-1 virions in a manner homologous to human tetherin, indicating that structure, rather than sequence, is the critical factor in tetherin function. The coiled-coil domain of tetherin contains a number of destabilising residues [147] that may provide tetherin with a high level of flexibility in order to facilitate tethering during budding. Mutations that disrupt the coiled-coil arrangement of the ectodomain prevent tetherin from functioning [148]. In 2013, Venkatesh and Bieniasz addressed possible tetherin configurations, finding that tetherin monomers form parallel dimers during entrapment of HIV-1 particles rather than antiparallel dimers [145]. Although tethered virions were found to be bound by tetherin in states where tetherin was anchored to the host cell by both the N- and C-terminus, the orientation whereby the tetherin N-terminal transmembrane domain is anchored in the cell membrane predominates [145]. Nevertheless, deletion of either terminal domain prevents tetherin functioning, indicating that either domain can interact with the viral envelope.

Viral antagonism of tetherin was first characterised using HIV-1 [136] and has since been observed in a number of viral pathogens; Kaposi’s sarcoma-associated herpesvirus, HIV-2, SIV, Chikungunya virus and Sendai virus all remove tetherin from the cell surface in a manner similar to HIV-1, with this process being initiated by Vpu, K5, Env, Nef, nsP1 and viral glycoproteins, respectively [149,150,151,152]. Tetherin counteraction by *Ebolavirus* has been demonstrated in 293T, HT1080 and HeLa cell lines by full-length eGP through interactions at the tetherin transmembrane domain [153]. eGP is also capable of rescuing HIV particles from restriction by human tetherin [153], indicating that this protein is unusually diverse in its antagonism of viruses.

eGP appears to be unique in its mechanism of inhibition compared to other viral antagonists of tetherin such as HIV Vpu, since expression of this protein does not change the amount of tetherin present at the cell surface [153,154]. In addition, eGP does not alter the localisation profile of tetherin by removing it from lipid rafts, nor does it inhibit the incorporation of tetherin into virion envelope membranes [138]. Retention in the endoplasmic reticulum inhibits eGP antagonism, and co-immunoprecipitation analysis shows that tetherin interacts exclusively with lower molecular mass forms of eGP, speculated to be immaturely glycosylated GP_1,2_ [153]. Additionally, mutations to the eGP transmembrane domain, which alter its correct localisation at the cell surface, also negatively impacts on the ability of eGP to block tetherin function [155]. Together, these findings indicate that surface-bound eGP functions in the late stages of its maturation at the cell surface to target tetherin.

In 2015, Gustin et al. reported that tetherin interacts with eVP40 in the absence of eGP [154], suggesting that this interaction may mediate tethering. When eVP40 is co-expressed with eGP, eVP40-tetherin interactions are inhibited [154]. These findings provide preliminary evidence that eGP may function by steric hindrance, preventing eVP40 interactions with tetherin. A GXXXA motif within the transmembrane domain of eGP is required for eGP inhibition of tetherin [156]. Mutations to the GXXXA motif result in decreased VLP egress from cells expressing tetherin, and an overall reduction in viral titres by 10-fold [156]. The specific role that the GXXXA motif plays in tetherin antagonism is yet to be elucidated and should be considered a priority for further investigation.

### 4.2. ISG15 Interferes with Nedd4 Ubiquitination of VP40

ISG15 (Interferon stimulated gene 15), like tetherin, is a broad-spectrum antiviral protein induced by type I IFN [157]. The antiviral properties of ISG15 rely on a ubiquitination-like mechanism (ISGylation), which conjugates ISG15 to a target protein at lysine rich residues, thereby altering the stability or function of the target in what is usually a regulatory role [158]. The expression of ISG15 during EBOV VLP budding significantly decreases eVP40 ubiquitination, the quantity of eVP40 present in VLPs, and budding efficiency, which is normally promoted by Nedd4 ubiquitination of eVP40 [159]. Evidence supports an interaction between ISG15 and Nedd4 rather than targeting of eVP40 itself, preventing Nedd4 from participating in normal interactions with eVP40. Co-immunoprecipitation of Nedd4 with ISG15 has been observed [160], and co-transfection of cells with plasmids expressing Nedd4, ISG15 and eVP40 significantly decreases levels of free ISG15 when compared to cells expressing ISG15 and eVP40 alone [159]. However, inhibition of budding by ISG15 is not observed in the context of the eVP40 L-domain mutant, which does not interact with Nedd4, or when ISG15 expression is knocked down by siRNAs, indicating that the presence of both ISG15 and the wild-type VP40 are necessary for inhibition to occur [159]. Conversely, treating cells with type I IFN increases budding inhibition, and is associated with ISG15 over-expression [159]. The presence of ISG15 prevents interactions between Nedd4 and the E2 enzymes required to promote the ubiquitination function of Nedd4 [160]. This finding indicates that the mechanism of ISG15 inhibition is through competition with E2 binding to Nedd4, and as a consequence this prevents Nedd4 promotion of VLP budding [160]. The potential interference of ISG15 on MARV budding has not been explored.

Influenza B, SARS-CoV, and Vaccinia viruses are all reported to escape the inhibitory effects of ISG15 through unique mechanisms involving NS1, papain-like protease (PLpro), and E3, respectively [161,162,163]. In these cases, viral antagonists prevent ISG15 from performing its homeostatic role in protein production [161,162,163]. However, there are no reports of a viral antagonist that targets ISG15 to restore EBOV budding. Nevertheless, the precedent set by Influenza B, SARS-CoV, and Vaccinia viruses makes it an intriguing possibility worthy of further investigation.

### 4.3. Bag3, A Newly Identified Inhibitor of Filovirus Budding

A third host inhibitor of filovirus budding, Bag3, was identified by a screen of proteins that interact with the PPxY motif of eVP40 [50]. Bag3 is a molecular co-chaperone that plays a homeostatic role in cytoskeleton protein expression and degradation of aggregations of misfolded proteins (aggresomes) [164]. Bag3 contains four unique domain types, with the N-terminal WW domain being critical for its interaction with VP40 [50]. Co-transfection assays in which wild-type Bag3 or eVP40 are replaced with Bag3 mutants lacking the N-terminal domain, or eVP40 PPxY L-domain deletion mutants nullify the interaction between these two proteins [50]. Expression of Bag3 during VLP and virus budding significantly decreases the egress of both EBOV and MARV particles in a dose-dependent manner, an effect that is reversed by Bag3-specific siRNA knockdown in VLP assays [50]. Although the mode of Bag3 inhibition is yet to be fully elucidated, preliminary evidence suggests that its function relies on a mechanism consistent with its homeostatic role in chaperone-based aggresome targeting and autophagy. In this regard, Bag3 re-localises VP40 away from the plasma membrane to sites that likely represent cellular aggresomes [50]. It is proposed that Bag3 is an EBOV and MARV VP40 specific host defense factor [50].

### 4.4. Application of Egress Inhibitors in Drug Development

Understanding the mechanisms by which filovirus egress is promoted and inhibited has proven useful in the identification of molecules with the potential to be explored as drug leads for a therapeutic agent for filoviruses. A number of small quinoxaline-based molecules have been identified as targeting the PPxY domain and are being investigated as potential leads [165]. These quinoxaline analogues inhibit EBOV and MARV VLPs, as well as a live recombinant VSV expressing the eVP40 PPxY L-domain at low nanomolar concentrations, and with little to no cytotoxicity [165]. In a similar study, a screen of candidate compounds was performed to identify small molecules that can competitively interfere with the PPxY/WW interaction between Nedd4 and VP40 (161). The presence of a conserved PPxY domain in a number of RNA viruses provides a target for a broad-spectrum antiviral therapeutic drug. Promising lead compounds from this screen have demonstrated broad-spectrum inhibition of the budding of enveloped viruses including filoviruses as well as arenaviruses and rhabdoviruses [166].

Additionally, knockdown of Vps4 expression by administration of a morpholino, an oligomer designed to modify gene expression, protects 70% of mice from challenges with lethal doses of EBOV [59], indicating that this protein may be a valuable target for drug design. Human cell-permeable antibodies (transbodies) specific to eVP40 have been generated as a preliminary attempt at a cell-penetrable immune-based treatment agent [167]. These transbodies, determined by indirect ELISA to interact with a cationic patch of the eVP40 C-terminal domain as well as the L-domain, effectively inhibited egress of EBOV VLPs [167].

## 5. Conclusions

Filovirus budding from host cells is a complex process, much of which is orchestrated through interactions with hijacked cellular proteins whose functions are applied in new ways in order to benefit the invading virus. Understanding the mechanisms by which host cell factors promote filovirus egress may reveal novel processes that could be exploited as new drug targets. The development of host countermeasures such as tetherin and ISG15, which interfere with filoviral budding, and indeed those measures that have been developed by the virus to counteract these inhibitors, is evidence for the complex “Red Queen effect” acting in this context. Although our understanding of the interaction between filoviruses and human hosts during virus egress is continually developing, there is undoubtedly more to learn about this process and the ways in which it can be exploited to advance prevention and treatment strategies against these viral pathogens.

## Figures and Tables

**Table 1:** Amino acid residues identified in EBOV VP40 as being necessary for efficient virion egress.

Location	Function
P7	Ubiquitination [42]
10PP11	Ubiquitination [42]
Y13	Ubiquitination [42]
P53	Membrane localisation [57]
A55	Dimerisation [51]
H61	Dimerisation [51]
W95	Oligomerisation [55]
96LPLGVA101	Membrane localisation and structural stability [62]
P108	Dimerisation [51]
112TA113	Dimerisation [51]
116ML117	Dimerisation [51]
F134	Oligomerisation [57]
A149	Oligomerisation [57]
A151	Oligomerisation [57]
E160	Oligomerisation [57]
Q184	Oligomerisation [57]
L203	Hexamerisation [51]
212KLR214	Structural stability and oligomerisation [40]
224KK225	Membrane localisation and membrane binding [51]
226GNSADLTSPE255	Protection of polymerised microtubules [63]
I237	Hexamerisation [51]
M241	Hexamerisation [51]
K270	Membrane localisation and binding [51]
274KK275	Membrane localisation and binding [51]
P283	Membrane binding [64]
P286	Membrane binding [64]
I293	Membrane penetration [58]
L295	Membrane penetration [58]
V298	Membrane penetration [58]
A299	Membrane penetration [65]
303LTMVI307	Hexamerisation [66]
309QDCDTCHSP317	Membrane binding [65]
320LPAVIEK326	Hexamerisation [67]

## References

[1] Brainard J., Hooper L., Pond K., Edmunds K., Hunter P.R. (2016). Risk factors for transmission of Ebola or Marburg virus disease: A systematic review and meta-analysis. Int. J. Epidemiol..

[2] Towner J.S., Amman B.R., Sealy T.K., Reeder Carroll S.A., Comer J.A., Kemp A., Swanepoel R., Paddock C.D., Balinandi S., Khristova M.L. (2009). Isolation of genetically diverse Marburg viruses from Egyptian fruit bats. PLoS Pathog..

[3] Leroy E.M., Kumulungui B., Pourrut X., Rouquet P., Hassanin A., Yaba P., Délicat A., Paweska J.T., Gonzalez J.P., Swanepoel R. (2005). Fruit bats as reservoirs of Ebola virus. Nature.

[4] Goldstein T., Anthony S.J., Gbakima A., Bird B.H., Bangura J., Tremeau-Bravard A., Belaganahalli M.N., Wells H.L., Dhanota J.K., Liang E. (2018). The discovery of Bombali virus adds further support for bats as hosts of ebolaviruses. Nat. Microbiol..

[5] Towner J.S., Khristova M.L., Sealy T.K., Vincent M.J., Erickson B.R., Bawiec D.A., Hartman A.L., Comer J.A., Zaki S.R., Stroher U. (2006). Marburgvirus genomics and association with a large hemorrhagic fever outbreak in Angola. J. Virol..

[6] De Clercq E. (2015). Ebola virus (EBOV) infection: Therapeutic strategies. Biochem. Pharmacol..

[7] Henao-Restrepo A.M., Camacho A., Longini I.M., Watson C.H., Edmunds W.J., Egger M., Carroll M.W., Dean N.E., Diatta I., Doumbia M. (2017). Efficacy and effectiveness of an rVSV-vectored vaccine in preventing Ebola virus disease: Final results from the Guinea ring vaccination, open-label, cluster-randomised trial (Ebola Ca Suffit!). Lancet.

[8] The PREVAIL II Writing Group, for the Multi-National PREVAIL II Study Team (2016). A randomized, controlled trial of ZMapp for Ebola virus infection. N. Engl. J. Med..

[9] Pavot V. (2016). Ebola virus vaccines: Where do we stand?. Clin. Immunol..

[10] Feldmann H., Klenk H.D., Sanchez A. (1993). Molecular biology and evolution of filoviruses. Arch. Virol. Suppl..

[11] Martin B., Hoenen T., Canard B., Decroly E. (2016). Filovirus proteins for antiviral drug discovery: A structure/function analysis of surface glycoproteins and virus entry. Antiviral Res..

[12] Whelan S.P.J., Barr J.N., Wertz G.W. (2004). Transcription and replication of nonsegmented negative-strand RNA viruses. Curr. Top. Microbiol. Immunol..

[13] Licata J.M., Johnson R.F., Han Z., Harty R.N. (2004). Contribution of Ebola virus glycoprotein, nucleoprotein, and VP24 to budding of VP40 virus-like particles. J. Virol..

[14] Sanchez A., Trappier S.G., Mahy B.W.J., Peters C.J., Nichol S.T. (1996). The virion glycoproteins of Ebola viruses are encoded in two reading frames and are expressed through transcriptional editing. Proc. Natl. Acad. Sci. USA.

[15] Volchkov V.E., Feldmann H., Volchkova V.A., Klenk H.D. (1998). Processing of the Ebola virus glycoprotein by the proprotein convertase furin. Proc. Natl. Acad. Sci. USA.

[16] Wool-Lewis R.J., Bates P. (1998). Characterization of Ebola virus entry by using pseudotyped viruses: Identification of receptor-deficient cell lines. J. Virol..

[17] Dolnik O., Volchkova V., Garten W., Carbonnelle C., Becker S., Kahnt J., Stroher U., Klenk H.D., Volchkov V. (2004). Ectodomain shedding of the glycoprotein GP of Ebola virus. EMBO J..

[18] Mehedi M., Falzarano D., Seebach J., Hu X., Carpenter M.S., Schnittler H.-J., Feldmann H. (2011). A new Ebola virus nonstructural glycoprotein expressed through RNA editing. J. Virol..

[19] Cook J.D., Lee J.E. (2013). The secret life of viral entry glycoproteins: Moonlighting in immune evasion. PLoS Pathog..

[20] Davey R.A., Shtanko O., Anantpadma M., Sakurai Y., Chandran K., Maury W. (2017). Mechanisms of filovirus entry. Curr. Top. Microbiol. Immunol..

[21] Herbert A.S., Davidson C., Kuehne A.I., Bakken R., Braigen S.Z., Gunn K.E., Whelan S.P., Brummelkamp T.R., Twenhafel N.A., Chandran K. (2015). Niemann-Pick C1 is essential for Ebolavirus replication and pathogenesis. mBio.

[22] Nanbo A., Imai M., Watanabe S., Noda T., Takahashi K., Neumann G., Halfmann P., Kawaoka Y. (2010). Ebolavirus is internalized into host cells via macropinocytosis in a viral glycoprotein-dependent manner. PLoS Pathog..

[23] Hood C.L., Abraham J., Boyington J.C., Leung K., Kwong P.D., Nabel G.J. (2010). Biochemical and structural characterization of cathepsin L-processed Ebola virus glycoprotein: implications for viral entry and immunogenicity. J. Virol..

[24] Chandran K., Sullivan N.J., Felbor U., Whelan S.P., Cunningham J.M. (2005). Endosomal proteolysis of the Ebola virus glycoprotein is necessary for infection. Science.

[25] Kaletsky R.L., Simmons G., Bates P. (2007). Proteolysis of the Ebola virus glycoproteins enhances virus binding and infectivity. J. Virol..

[26] Schornberg K., Matsuyama S., Kabsch K., Delos S., Bouton A., White J. (2006). Role of endosomal cathepsins in entry mediated by the Ebola virus glycoprotein. J. Virol..

[27] Brecher M., Schornberg K.L., Delos S.E., Fusco M.L., Saphire E.O., White J.M. (2012). Cathepsin cleavage potentiates the ebola virus glycoprotein to undergo a subsequent fusion-relevant conformational change. J. Virol..

[28] Weik M., Modrof J., Klenk H., Becker S., Mühlberger E. (2002). Ebola virus VP30-mediated transcription is regulated by RNA secondary structure formation. J. Virol..

[29] Muhlberger E., Lotfering B., Klenk H., Becker S. (1998). Three of the Four Nucleocapsid Proteins of Marburg Virus, NP, VP35, and L, Are Sufficient To Mediate Replication and Transcription of Marburg Virus-Specific Monocistronic Minigenomes. J. Virol..

[30] Hoenen T., Shabman R.S., Groseth A., Herwig A., Weber M., Schudt G., Dolnik O., Basler C.F., Becker S., Feldmann H. (2012). Inclusion bodies are a site of ebolavirus replication. J. Virol..

[31] Nanbo A., Watanabe S., Halfmann P., Kawaoka Y. (2013). The spatio-temporal distribution dynamics of Ebola virus proteins and RNA in infected cells. Sci. Rep..

[32] Bavari S., Bosio C.M., Wiegand E., Ruthel G., Will A.B., Geisbert T.W., Hevey M., Schmaljohn C., Schmaljohn A., Aman M.J. (2002). Lipid raft microdomains: A gateway for compartmentalized trafficking of Ebola and Marburg viruses. J. Exp. Med..

[33] Elliott L.H., Kiley M.P., McCormick J.B. (1985). Descriptive analysis of Ebola virus proteins. Virology.

[34] Geisbert T.W., Jahrling P.B. (1995). Differentiation of filoviruses by electron microscopy. Virus Res..

[35] Noda T., Sagara H., Suzuki E., Takada A., Kida H., Kawaoka Y. (2002). Ebola virus VP40 drives the formation of virus-like filamentous particles along with GP. J. Virol..

[36] Noda T., Watanabe S., Sagara H., Kawaoka Y. (2007). Mapping of the VP40-binding regions of the nucleoprotein of Ebola virus. J. Virol..

[37] Timmins J., Scianimanico S., Schoehn G., Weissenhorn W. (2001). Vesicular release of Ebola virus matrix protein VP40. Virology.

[38] Wenigenrath J., Kolesnikova L., Hoenen T., Mittler E., Becker S. (2010). Establishment and application of an infectious virus-like particle system for Marburg virus. J. Gen. Virol..

[39] Oda S., Noda T., Wijesinghe K.J., Halfmann P., Bornholdt Z.A., Abelson D.M., Armbrust T., Stahelin R.V., Kawaoka Y., Saphire E.O. (2015). Crystal structure of Marburg virus VP40 reveals a broad, basic patch for matrix assembly and a requirement of the N-terminal domain for immunosuppression. J. Virol..

[40] McCarthy S.E., Johnson R.F., Zhang Y.A., Sunyer J.O., Harty R.N. (2007). Role for amino acids 212KLR214 of Ebola virus VP40 in assembly and budding. J. Virol..

[41] Licata J.M., Simpson-Holley M., Wright N.T., Han Z., Paragas J., Harty R.N. (2003). Overlapping motifs (PTAP and PPEY) within the Ebola virus VP40 protein function independently as late budding domains: Involvement of host proteins TSG101 and VPS-4. J. Virol..

[42] Martin-Serrano J., Perez-Caballero D., Bieniasz P.D. (2004). Context-dependent effects of L domains and ubiquitination on viral budding. J. Virol..

[43] Jasenosky L.D., Neumann G., Lukashevich I., Kawaoka Y. (2001). Ebola virus VP40-induced particle formation and association with the lipid bilayer. J. Virol..

[44] Neumann G., Ebihara H., Takada A., Noda T., Kobasa D., Jasenosky L.D., Watanabe S., Kim J.H., Feldmann H., Kawaoka Y. (2005). Ebola virus VP40 late domains are not essential for viral replication in cell culture. J. Virol..

[45] Urata S., Yasuda J. (2010). Regulation of Marburg virus (MARV) budding by Nedd4.1: A different WW domain of Nedd4.1 is critical for binding to MARV and Ebola virus VP40. J. Gen. Virol..

[46] Makino A., Yamayoshi S., Shinya K., Noda T., Kawaoka Y. (2011). Identification of amino acids in Marburg virus VP40 that are important for virus-like particle budding. J. Infect. Dis..

[47] Chen H.I., Sudol M. (1995). The WW domain of Yes-associated protein binds a proline-rich ligand that differs from the consensus established for Src homology 3-binding modules. Proc. Natl. Acad. Sci. USA.

[48] Harvey K.F., Kumar S. (1999). Nedd4-like proteins: An emerging family of ubiquitin-protein ligases implicated in diverse cellular functions. Trends Cell Biol..

[49] Han Z., Sagum C.A., Bedford M.T., Sidhu S.S., Sudol M., Harty R.N. (2016). ITCH E3 ubiquitin ligase interacts with Ebola virus VP40 to regulate budding. J. Virol..

[50] Liang J., Sagum C.A., Bedford M.T., Sidhu S.S., Sudol M., Han Z., Harty R.N. (2017). Chaperone-mediated autophagy protein BAG3 negatively regulates Ebola and Marburg VP40-mediated egress. PLoS Pathog..

[51] Bornholdt Z.A., Noda T., Abelson D.M., Halfmann P., Wood M.R., Kawaoka Y., Saphire E.O. (2013). Structural rearrangement of Ebola virus VP40 begets multiple functions in the virus life cycle. Cell.

[52] Timmins J., Schoehn G., Kohlhaas C., Klenk H.D., Ruigrok R.W., Weissenhorn W. (2003). Oligomerization and polymerization of the filovirus matrix protein VP40. Virology.

[53] Hoenen T., Volchkov V., Kolesnikova L., Mittler E., Timmins J., Ottmann M., Reynard O., Becker S., Weissenhorn W. (2005). VP40 octamers are essential for Ebola virus replication. J. Virol..

[54] Gomis-Rüth F.X., Dessen A., Timmins J., Bracher A., Kolesnikowa L., Becker S., Klenk H.-D., Weissenhorn W. (2003). The matrix protein VP40 from Ebola virus octamerizes into pore-like structures with specific RNA binding properties. Structure.

[55] Hoenen T., Biedenkopf N., Zielecki F., Jung S., Groseth A., Feldmann H., Becker S. (2010). Oligomerization of Ebola virus VP40 is essential for particle morphogenesis and regulation of viral transcription. J. Virol..

[56] Gc J.B., Gerstman B.S., Stahelin R.V., Chapagain P.P. (2016). The Ebola virus protein VP40 hexamer enhances the clustering of PI(4,5)P2 lipids in the plasma membrane. Phys. Chem. Chem. Phys..

[57] Yamayoshi S., Kawaoka Y. (2007). Mapping of a region of Ebola virus VP40 that is important in the production of virus-like particles. J. Infect. Dis..

[58] Adu-Gyamfi E., Soni S.P., Xue Y., Digman M.A., Gratton E., Stahelin R.V. (2013). The Ebola virus matrix protein penetrates into the plasma membrane: A key step in viral protein 40 (VP40) oligomerization and viral egress. J. Biol. Chem..

[59] Silvestri L.S., Ruthel G., Kallstrom G., Warfield K.L., Swenson D.L., Nelle T., Iversen P.L., Bavari S., Aman M.J. (2007). Involvement of vacuolar protein sorting pathway in Ebola virus release independent of TSG101 interaction. J. Infect. Dis..

[60] Adu-Gyamfi E., Soni S.P., Jee C.S., Digman M.A., Gratton E., Stahelin R.V. (2014). A loop region in the N-terminal domain of Ebola virus VP40 is important in viral assembly, budding, and egress. Viruses.

[61] Liu Y., Cocka L., Okumura A., Zhang Y.A., Sunyer J.O., Harty R.N. (2010). Conserved motifs within Ebola and Marburg virus VP40 proteins are important for stability, localization, and subsequent budding of virus-like particles. J. Virol..

[62] Liu Y., Harty R.N. (2010). Viral and host proteins that modulate filovirus budding. Future Virol..

[63] Ruthel G., Demmin G.L., Kallstrom G., Javid M.P., Badie S.S., Will A.B., Nelle T., Schokman R., Nguyen T.L., Carra J.H. (2005). Association of ebola virus matrix protein VP40 with microtubules. J. Virol..

[64] Panchal R.G., Ruthel G., Kenny T.A., Kallstrom G.H., Lane D., Badie S.S., Li L., Bavari S., Aman M.J. (2003). In vivo oligomerization and raft localization of Ebola virus protein VP40 during vesicular budding. Proc. Natl. Acad. Sci. USA.

[65] Soni S.P., Adu-Gyamfi E., Yong S.S., Jee C.S., Stahelin R.V. (2013). The Ebola virus matrix protein deeply penetrates the plasma membrane: An important step in viral egress. Biophys. J..

[66] Yamayoshi S., Noda T., Ebihara H., Goto H., Morikawa Y., Lukashevich I.S., Neumann G., Feldmann H., Kawaoka Y. (2008). Ebola virus matrix protein VP40 uses the COPII transport system for its intracellular transport. Cell Host Microbe.

[67] Scianimanico S., Schoehn G., Timmins J., Ruigrok R.H., Klenk H.D., Weissenhorn W. (2000). Membrane association induces a conformational change in the Ebola virus matrix protein. EMBO J..

[68] Soni S.P., Stahelin R.V. (2014). The Ebola virus matrix protein VP40 selectively induces vesiculation from phosphatidylserine-enriched membranes. J. Biol. Chem..

[69] Rog T., Pasenkiewicz-Gierula M. (2001). Cholesterol effects on the phospholipid condensation and packing in the bilayer: A molecular simulation study. FEBS Lett..

[70] Wijesinghe K.J., Stahelin R.V. (2015). Investigation of the lipid binding properties of the Marburg virus matrix protein VP40. J. Virol..

[71] de Meyer F., Smit B. (2009). Effect of cholesterol on the structure of a phospholipid bilayer. Proc. Natl. Acad. Sci. USA.

[72] Dick R.A., Goh S.L., Feigenson G.W., Vogt V.M. (2012). HIV-1 Gag protein can sense the cholesterol and acyl chain environment in model membranes. Proc. Natl. Acad. Sci. USA.

[73] Adu-Gyamfi E., Johnson K.A., Fraser M.E., Scott J.L., Soni S.P., Jones K.R., Digman M.A., Gratton E., Tessier C.R., Stahelin R.V. (2015). Host cell plasma membrane phosphatidylserine regulates the assembly and budding of Ebola virus. J. Virol..

[74] Dessen A., Volchkov V., Dolnik O., Klenk H.D., Weissenhorn W. (2000). Crystal structure of the matrix protein VP40 from Ebola virus. EMBO J..

[75] Del Vecchio K., Frick C.T., Gc J.B., Oda S.-I., Gerstman B.S., Saphire E.O., Chapagain P.P., Stahelin R.V. (2018). A cationic, C-terminal patch and structural rearrangements in Ebola virus matrix VP40 protein control its interactions with phosphatidylserine. J. Biol. Chem..

[76] Shnyrova A.V., Ayllon J., Mikhalyov I.I., Villar E., Zimmerberg J., Frolov V.A. (2007). Vesicle formation by self-assembly of membrane-bound matrix proteins into a fluidlike budding domain. J. Cell Biol..

[77] Johnson K.A., Taghon G.J., Scott J.L., Stahelin R.V. (2016). The Ebola virus matrix protein, VP40, requires phosphatidylinositol 4,5-bisphosphate (PI(4,5)P2) for extensive oligomerization at the plasma membrane and viral egress. Sci. Rep..

[78] Monde K., Chukkapalli V., Ono A. (2011). Assembly and replication of HIV-1 in T cells with low levels of phosphatidylinositol-(4,5)-bisphosphate. J. Virol..

[79] Swenson D.L., Warfield K.L., Kuehl K., Larsen T., Hevey M.C., Schmaljohn A., Bavari S., Aman M.J. (2004). Generation of Marburg virus-like particles by co-expression of glycoprotein and matrix protein. FEMS Immunol. Med. Microbiol..

[80] Urata S., Noda T., Kawaoka Y., Morikawa S., Yokosawa H., Yasuda J. (2007). Interaction of Tsg101 with Marburg virus VP40 depends on the PPPY motif, but not the PT/SAP motif as in the case of Ebola virus, and Tsg101 plays a critical role in the budding of Marburg virus-like particles induced by VP40, NP, and GP. J. Virol..

[81] Dolnik O., Kolesnikova L., Stevermann L., Becker S. (2010). Tsg101 is recruited by a late domain of the nucleocapsid protein to support budding of Marburg virus-like particles. J. Virol..

[82] Wahl-Jensen V.M., Afanasieva T.A., Seebach J., Ströher U., Feldmann H., Schnittler H.-J. (2005). Effects of Ebola virus glycoproteins on endothelial cell activation and barrier function. J. Virol..

[83] Han Z., Harty R.N. (2005). Packaging of actin into Ebola virus VLPs. Virol. J..

[84] Kolesnikova L., Bohil A.B., Cheney R.E., Becker S. (2007). Budding of Marburgvirus is associated with filopodia. Cell. Microbiol..

[85] Adu-Gyamfi E., Digman M.A., Gratton E., Stahelin R.V. (2012). Single-particle tracking demonstrates that actin coordinates the movement of the Ebola virus matrix protein. Biophys. J..

[86] Martinez N.W., Xue X., Berro R.G., Kreitzer G., Resh M.D. (2008). Kinesin KIF4 regulates intracellular trafficking and stability of the human immunodeficiency virus type 1 Gag polyprotein. J. Virol..

[87] Radtke K., Kieneke D., Wolfstein A., Michael K., Steffen W., Scholz T., Karger A., Sodeik B. (2010). Plus- and minus-end directed microtubule motors bind simultaneously to herpes simplex virus capsids using different inner tegument structures. PLoS Pathog..

[88] Ward B.M. (2005). Visualization and characterization of the intracellular movement of Vaccinia virus intracellular mature virions. J. Virol..

[89] Kolesnikova L., Bugany H., Klenk H.D., Becker S. (2002). VP40, the matrix protein of Marburg virus, is associated with membranes of the late endosomal compartment. J. Virol..

[90] Hicke L. (2001). Protein regulation by monoubiquitin. Nat. Rev. Mol. Cell Biol..

[91] d’Azzo A., Bongiovanni A., Nastasi T. (2005). E3 ubiquitin ligases as regulators of membrane protein trafficking and degradation. Traffic.

[92] Anan T., Nagata Y., Koga H., Honda Y., Yabuki N., Miyamoto C., Kuwano A., Matsuda I., Endo F., Saya H. (1998). Human ubiquitin-protein ligase Nedd4: Expression, subcellular localization and selective interaction with ubiquitin-conjugating enzymes. Genes Cells.

[93] Lafont F., Simons K. (2001). Raft-partitioning of the ubiquitin ligases Cbl and Nedd4 upon IgE-triggered cell signaling. Proc. Natl. Acad. Sci. USA.

[94] Yasuda J., Nakao M., Kawaoka Y., Shida H. (2003). Nedd4 regulates egress of Ebola virus-like particles from host cells. J. Virol..

[95] Angers A., Ramjaun A.R., McPherson P.S. (2004). The HECT domain ligase Itch ubiquitinates endophilin and localizes to the trans-Golgi network and endosomal system. J. Biol. Chem..

[96] Scheffner M., Kumar S. (2014). Mammalian HECT ubiquitin-protein ligases: Biological and pathophysiological aspects. Biochim. Biophys. Acta.

[97] Gallagher E., Gao M., Liu Y.-C., Karin M. (2006). Activation of the E3 ubiquitin ligase Itch through a phosphorylation-induced conformational change. Proc. Natl. Acad. Sci. USA.

[98] Han Z., Sagum C.A., Takizawa F., Ruthel G., Berry C.T., Kong J., Sunyer J.O., Freedman B.D., Bedford M.T., Sidhu S.S. (2017). Ubiquitin ligase WWP1 interacts with Ebola virus VP40 to regulate egress. J. Virol..

[99] Kershaw N.J., Laktyushin A., Nicola N.A., Babon J.J. (2014). Reconstruction of an active SOCS3-based E3 ubiquitin ligase complex in vitro: Identification of the active components and JAK2 and gp130 as substrates. Growth Factors.

[100] Babon J.J., Sabo J.K., Soetopo A., Yao S., Bailey M.F., Zhang J.G., Nicola N.A., Norton R.S. (2008). The SOCS box domain of SOCS3: Structure and interaction with the elonginBC-cullin5 ubiquitin ligase. J. Mol. Biol..

[101] Okumura A., Rasmussen A.L., Halfmann P., Feldmann F., Yoshimura A., Feldmann H., Kawaoka Y., Harty R.N., Katze M.G. (2015). Suppressor of cytokine signaling 3 is an inducible host factor that regulates virus egress during Ebola virus infection. J. Virol..

[102] Yan C., Cao J., Wu M., Zhang W., Jiang T., Yoshimura A., Gao H. (2010). Suppressor of cytokine signaling 3 inhibits LPS-induced IL-6 expression in osteoblasts by suppressing CCAAT/enhancer-binding protein [102] activity. J. Biol. Chem..

[103] Henne W.M., Buchkovich N.J., Emr S.D. (2011). The ESCRT Pathway. Dev. Cell.

[104] Goff A., Ehrlich L.S., Cohen S.N., Carter C.A. (2003). Tsg101 control of human immunodeficiency virus type 1 Gag trafficking and release. J. Virol..

[105] Patton G.S., Morris S.A., Chung W., Bieniasz P.D., McClure M.O. (2005). Identification of domains in Gag important for prototypic foamy virus egress. J. Virol..

[106] Segura-Morales C., Pescia C., Chatellard-Causse C., Sadoul R., Bertrand E., Basyuk E. (2005). Tsg101 and Alix interact with murine leukemia virus Gag and cooperate with Nedd4 ubiquitin ligases during budding. J. Biol. Chem..

[107] Perez M., Craven R.C., de la Torre J.C. (2003). The small RING finger protein Z drives arenavirus budding: Implications for antiviral strategies. Proc. Natl. Acad. Sci. USA.

[108] Dolnik O., Kolesnikova L., Welsch S., Strecker T., Schudt G., Becker S. (2014). Interaction with Tsg101 is necessary for the efficient transport and release of nucleocapsids in Marburg virus-infected cells. PLoS Pathog..

[109] Martin-Serrano J., Zang T., Bieniasz P.D. (2001). HIV-1 and Ebola virus encode small peptide motifs that recruit Tsg101 to sites of particle assembly to facilitate egress. Nat. Med..

[110] Morita E., Sandrin V., Chung H.Y., Morham S.G., Gygi S.P., Rodesch C.K., Sundquist W.I. (2007). Human ESCRT and ALIX proteins interact with proteins of the midbody and function in cytokinesis. EMBO J..

[111] Razi M., Futter C.E. (2006). Distinct Roles for Tsg101 and Hrs in multivesicular body formation and inward vesiculation. Mol. Biol. Cell.

[112] Pornillos O., Alam S.L., Rich R.L., Myszka D.G., Davis D.R., Sundquist W.I. (2002). Structure and functional interactions of the Tsg101 UEV domain. EMBO J..

[113] Irie T., Licata J.M., Harty R.N. (2005). Functional characterization of Ebola virus L-domains using VSV recombinants. Virology.

[114] Lu J., Qu Y., Liu Y., Jambusaria R., Han Z., Ruthel G., Freedman B.D., Harty R.N. (2013). Host IQGAP1 and Ebola virus VP40 interactions facilitate virus-like particle egress. J. Virol..

[115] Christ L., Wenzel E.M., Liestøl K., Raiborg C., Campsteijn C., Stenmark H. (2016). ALIX and ESCRT-I/II function as parallel ESCRT-III recruiters in cytokinetic abscission. J. Cell Biol..

[116] Dussupt V., Javid M.P., Abou-Jaoude G., Jadwin J.A., de La Cruz J., Nagashima K., Bouamr F. (2009). The nucleocapsid region of HIV-1 Gag cooperates with the PTAP and LYPXnL late domains to recruit the cellular machinery necessary for viral budding. PLoS Pathog..

[117] Bissig C., Gruenberg J. (2014). ALIX and the multivesicular endosome: ALIX in Wonderland. Trends Cell Biol..

[118] Han Z., Madara J.J., Liu Y., Liu W., Ruthel G., Freedman B.D., Harty R.N. (2015). ALIX rescues budding of a double PTAP/PPEY L-domain deletion mutant of Ebola VP40: A role for ALIX in Ebola virus egress. J. Infect. Dis..

[119] Zhai Q., Landesman M.B., Chung H.-Y., Dierkers A., Jeffries C.M., Trewhella J., Hill C.P., Sundquist W.I. (2011). Activation of the retroviral budding factor ALIX. J. Virol..

[120] Sette P., Jadwin J.A., Dussupt V., Bello N.F., Bouamr F. (2010). The ESCRT-associated protein Alix recruits the ubiquitin ligase Nedd4-1 to facilitate HIV-1 release through the LYPXnL L domain motif. J. Virol..

[121] Landsberg M.J., Vajjhala P.R., Rothnagel R., Munn A.L., Hankamer B. (2009). Three-dimensional structure of AAA ATPase Vps4: Advancing structural insights into the mechanisms of endosomal sorting and enveloped virus budding. Structure.

[122] Babst M., Wendland B., Estepa E.J., Emr S.D. (1998). The Vps4p AAA ATPase regulates membrane association of a Vps protein complex required for normal endosome function. EMBO J..

[123] Schoeneberg J., Yan S., Righini M., Remec Pavlin M., Lee I.-H., Carlson L.-A., Bahrami A.H., Goldman D.H., Ren X., Hummer G. (2018). ATP-dependent force generation and membrane scission by ESCRT-III and Vps4. bioRxiv.

[124] Kolesnikova L., Strecker T., Morita E., Zielecki F., Mittler E., Crump C., Becker S. (2009). Vacuolar protein sorting pathway contributes to the release of Marburg virus. J. Virol..

[125] Wang J., Peng Q., Lin Q., Childress C., Carey D., Yang W. (2010). Calcium activates Nedd4 E3 ubiquitin ligases by releasing the C2 domain-mediated auto-inhibition. J. Biol. Chem..

[126] Bissig C., Lenoir M., Velluz M.C., Kufareva I., Abagyan R., Overduin M., Gruenberg J. (2013). Viral infection controlled by a calcium-dependent lipid-binding module in ALIX. Dev. Cell.

[127] Scheffer L.L., Sreetama S.C., Sharma N., Medikayala S., Brown K.J., Defour A., Jaiswal J.K. (2014). Mechanism of Ca(2+)-triggered ESCRT assembly and regulation of cell membrane repair. Nat. Commun..

[128] Ehrlich L.S., Medina G.N., Carter C.A. (2011). ESCRT machinery potentiates HIV-1 utilization of the PI(4,5)P(2)-PLC-IP3R-Ca(2+) signaling cascade. J. Mol. Biol..

[129] Ehrlich L.S., Carter C.A. (2012). HIV assembly and budding: Ca(2+) signaling and non-ESCRT proteins set the stage. Mol. Biol. Int..

[130] Han Z., Harty R.N. (2007). Influence of calcium/calmodulin on budding of Ebola VLPs: Implications for the involvement of the Ras/Raf/MEK/ERK pathway. Virus Genes.

[131] Muik M., Fahrner M., Schindl R., Stathopulos P., Frischauf I., Derler I., Plenk P., Lackner B., Groschner K., Ikura M. (2011). STIM1 couples to ORAI1 via an intramolecular transition into an extended conformation. EMBO J..

[132] Han Z., Madara J.J., Herbert A., Prugar L.I., Ruthel G., Lu J., Liu Y., Liu W., Liu X., Wrobel J.E. (2015). Calcium regulation of hemorrhagic fever virus budding: Mechanistic implications for host-oriented therapeutic ontervention. PLoS Pathog..

[133] Neil S.J.D., Sandrin V., Sundquist W.I., Bieniasz P.D. (2007). An interferon-α-induced tethering mechanism inhibits HIV-1 and Ebola virus particle release but is counteracted by the HIV-1 Vpu protein. Cell Host Microbe.

[134] Radoshitzky S.R., Dong L., Chi X., Clester J.C., Retterer C., Spurgers K., Kuhn J.H., Sandwick S., Ruthel G., Kota K. (2010). Infectious Lassa virus, but not filoviruses, is restricted by BST-2/tetherin. J. Virol..

[135] Erikson E., Adam T., Schmidt S., Lehmann-Koch J., Over B., Goffinet C., Harter C., Bekeredjian-Ding I., Sertel S., Lasitschka F. (2011). In vivo expression profile of the antiviral restriction factor and tumor-targeting antigen CD317/BST-2/HM1.24/tetherin in humans. Proc. Natl. Acad. Sci. USA.

[136] Hammonds J., Wang J.J., Yi H., Spearman P. (2010). Immunoelectron microscopic evidence for Tetherin/BST2 as the physical bridge between HIV-1 virions and the plasma membrane. PLoS Pathog..

[137] Neil S.J., Zang T., Bieniasz P.D. (2008). Tetherin inhibits retrovirus release and is antagonized by HIV-1 Vpu. Nature.

[138] Klimkait T., Strebel K., Hoggan M.D., Martin M.A., Orenstein J.M. (1990). The human immunodeficiency virus type 1-specific protein vpu is required for efficient virus maturation and release. J. Virol..

[139] Lopez L.A., Yang S.J., Exline C.M., Rengarajan S., Haworth K.G., Cannon P.M. (2012). Anti-tetherin activities of HIV-1 Vpu and ebola virus glycoprotein do not involve removal of tetherin from lipid rafts. J. Virol..

[140] Neil S.J.D., Eastman S.W., Jouvenet N., Bieniasz P.D. (2006). HIV-1 Vpu promotes release and prevents endocytosis of nascent retrovirus particles from the plasma membrane. PLoS Pathog..

[141] Kuhl B.D., Cheng V., Wainberg M.A., Liang C. (2011). Tetherin and its viral antagonists. J. Neuroimmune Pharmacol..

[142] le Tortorec A., Willey S., Neil S.J.D. (2011). Antiviral inhibition of enveloped virus release by Tetherin/BST-2: Action and counteraction. Viruses.

[143] Kupzig S., Korolchuk V., Rollason R., Sugden A., Wilde A., Banting G. (2003). Bst-2/HM1.24 is a raft-associated apical membrane protein with an unusual topology. Traffic.

[144] Ishikawa J., Kaisho T., Tomizawa H., Lee B.O., Kobune Y., Inazawa J., Oritani K., Itoh M., Ochi T., Ishihara K. (1995). Molecular cloning and chromosomal mapping of a bone marrow stromal cell surface gene, BST2, that may be involved in pre-B-cell growth. Genomics.

[145] Venkatesh S., Bieniasz P.D. (2013). Mechanism of HIV-1 virion entrapment by tetherin. PLoS Pathog..

[146] Perez-Caballero D., Zang T., Ebrahimi A., McNatt M.W., Gregory D.A., Johnson M.C., Bieniasz P.D. (2009). Tetherin inhibits HIV-1 release by directly tethering virions to cells. Cell.

[147] Hinz A., Miguet N., Natrajan G., Usami Y., Yamanaka H., Renesto P., Hartlieb B., McCarthy A.A., Simorre J.P., Gottlinger H. (2010). Structural basis of HIV-1 tethering to membranes by the BST-2/tetherin ectodomain. Cell Host Microbe.

[148] Hammonds J., Ding L., Chu H., Geller K., Robbins A., Wang J.J., Yi H., Spearman P. (2012). The tetherin/BST-2 coiled-coil ectodomain mediates plasma membrane microdomain localization and restriction of particle release. J. Virol..

[149] Bampi C., Rasga L., Roux L. (2013). Antagonism to human BST-2/tetherin by Sendai virus glycoproteins. J. Gen. Virol..

[150] Jones P.H., Maric M., Madison M.N., Maury W., Roller R.J., Okeoma C.M. (2013). BST-2/tetherin-mediated restriction of chikungunya (CHIKV) VLP budding is counteracted by CHIKV non-structural protein 1 (nsP1). Virology.

[151] Hauser H., Lopez L.A., Yang S.J., Oldenburg J.E., Exline C.M., Guatelli J.C., Cannon P.M. (2010). HIV-1 Vpu and HIV-2 Env counteract BST-2/tetherin by sequestration in a perinuclear compartment. Retrovirology.

[152] Mansouri M., Viswanathan K., Douglas J.L., Hines J., Gustin J., Moses A.V., Früh K. (2009). Molecular mechanism of BST2/tetherin downregulation by K5/MIR2 of Kaposi’s sarcoma-associated herpesvirus. J. Virol..

[153] Bates P., Kaletsky R.L., Francica J.R., Agrawal-Gamse C. (2009). Tetherin-mediated restriction of filovirus budding is antagonized by the Ebola glycoprotein. Proc. Natl. Acad. Sci. USA.

[154] Gustin J.K., Bai Y., Moses A.V., Douglas J.L. (2015). Ebola virus glycoprotein promotes enhanced viral egress by preventing Ebola VP40 from associating with the host restriction factor BST2/tetherin. J. Infect. Dis..

[155] Gnirß K., Fiedler M., Krämer-Kühl A., Bolduan S., Mittler E., Becker S., Schindler M., Pöhlmann S. (2014). Analysis of determinants in filovirus glycoproteins required for tetherin antagonism. Viruses.

[156] González-Hernández M., Hoffmann M., Brinkmann C., Nehls J., Winkler M., Schindler M., Pöhlmann S. (2018). A GXXXA motif in the transmembrane domain of the Ebola virus glycoprotein is required for tetherin antagonism. J. Virol..

[157] Pitha-Rowe I.F., Pitha P.M. (2007). Viral defense, carcinogenesis and ISG15: Novel roles for an old ISG. Cytokine Growth Factor Rev..

[158] Zhao C., Denison C., Huibregtse J.M., Gygi S., Krug R.M. (2005). Human ISG15 conjugation targets both IFN-induced and constitutively expressed proteins functioning in diverse cellular pathways. Proc. Natl. Acad. Sci. USA.

[159] Okumura A., Pitha P.M., Harty R.N. (2008). ISG15 inhibits Ebola VP40 VLP budding in an l-domain-dependent manner by blocking Nedd4 ligase activity. Proc. Natl. Acad. Sci. USA.

[160] Malakhova O.A., Zhang D.-E. (2008). ISG15 inhibits Nedd4 ubiquitin E3 activity and enhances the innate antiviral response. J. Biol. Chem..

[161] Lindner H.A., Lytvyn V., Qi H., Lachance P., Ziomek E., Menard R. (2007). Selectivity in ISG15 and ubiquitin recognition by the SARS coronavirus papain-like protease. Arch. Biochem. Biophys..

[162] Yuan W., Krug R.M. (2001). Influenza B virus NS1 protein inhibits conjugation of the interferon (IFN)-induced ubiquitin-like ISG15 protein. EMBO J..

[163] Guerra S., Caceres A., Knobeloch K.P., Horak I., Esteban M. (2008). Vaccinia virus E3 protein prevents the antiviral action of ISG15. PLoS Pathog..

[164] Gamerdinger M., Kaya A.M., Wolfrum U., Clement A.M., Behl C. (2011). BAG3 mediates chaperone-based aggresome-targeting and selective autophagy of misfolded proteins. EMBO Rep..

[165] Loughran H.M., Han Z., Wrobel J.E., Decker S.E., Ruthel G., Freedman B.D., Harty R.N., Reitz A.B. (2016). Quinoxaline-based inhibitors of Ebola and Marburg VP40 egress. Bioorg. Med. Chem. Lett..

[166] Han Z., Lu J., Liu Y., Davis B., Lee M.S., Olson M.A., Ruthel G., Freedman B.D., Schnell M.J., Wrobel J.E. (2014). Small-molecule probes targeting the viral PPxY-host Nedd4 interface block egress of a broad range of RNA viruses. J. Virol..

[167] Teimoori S., Seesuay W., Jittavisutthikul S., Chaisri U., Sookrung N., Densumite J., Saelim N., Chulanetra M., Maneewatch S., Chaicumpa W. (2016). Human transbodies to VP40 inhibit cellular egress of Ebola virus-like particles. Biochem. Biophys. Res. Commun..

